# A Survey on the Current Status of Joubert Syndrome and Related Disorders Conducted Through a Patient and Family Group in Japan

**DOI:** 10.7759/cureus.112985

**Published:** 2026-07-19

**Authors:** Hiroshi Mano

**Affiliations:** 1 Rehabilitation Medicine, Shizuoka Children's Hospital, Shizuoka, JPN; 2 Rehabilitation Medicine, The University of Tokyo Hospital, Tokyo, JPN

**Keywords:** advocacy group, education, joubert syndrome, medical care, patient and family group, rare and intractable diseases, rehabilitation, social welfare

## Abstract

Introduction

Joubert syndrome and related disorders (JSRD) are rare and intractable diseases characterized by delayed psychomotor development, hypotonia and/or ataxia, and abnormal respiratory and eye movements. The Patient and Family Advocacy Group for Joubert Syndrome and Related Disorders in Japan was established in 2016. Since its inception, meetings for patients and families have been held approximately once a year.

Methods

An advocacy group meeting was held at our facility, consisting of a medical lecture and an open forum for information exchange among patients and families. A post-meeting questionnaire was administered to assess the needs and current circumstances of patients and families.

Results

Many patients were enrolled in or had attended special needs schools or received individualized educational accommodations. All patients had previously received rehabilitation therapy, with a significant proportion continuing therapy at the time of the survey. Families indicated a strong need for information on a range of topics, including medical care, social welfare, and education.

Conclusions

Addressing the ongoing needs of patients and families with rare and intractable diseases in the areas of healthcare, research, and support system remains a continuing challenge.

## Introduction

Joubert syndrome is a rare and intractable disease characterised by the agenesis of the cerebellar vermis, episodic hyperpnea, abnormal eye movements, ataxia, and psychomotor delay [1]. As a group of diseases that show "molar tooth sign" on cerebral magnetic resonance imaging, the concept of Joubert syndrome and related disorders (JSRD) has been proposed [2]. Various Joubert syndrome-related diseases, such as Arima syndrome [3], Senior-Loken syndrome [4,5], COACH (cerebellar vermis hypoplasia, oligophrenia (intellectual disability), ataxia, coloboma, and hepatic fibrosis) syndrome [6], and Dekabann syndrome [7], have been reported previously.

Although few studies on JSRD have been conducted in Japan, a research group under the Ministry of Health, Labour and Welfare was established in FY2011 to investigate these conditions, primarily through epidemiological studies led by Itoh et al. and supported by the Health and Labour Sciences Research Grants. In a nationwide two-stage survey on Arima syndrome conducted by Ito et al. in 2010 [8], 45 patients were identified in the primary survey. Of these, 23 were further evaluated in the secondary survey, with seven diagnosed with Arima syndrome and 16 with Joubert syndrome. While the prevalence of JSRD is estimated to be approximately 1 in 100,000 births in the United States and the Netherlands [9,10], precise data for Japan remain unavailable. However, a 2015 nationwide survey reported approximately 100 affected patients in Japan [11]. To date, over 40 genes associated with Joubert syndrome have been identified, with their phenotypic correlations increasingly recognized [12]. A genetic analysis by Suzuki et al. of 30 Japanese families revealed causative mutations in 25, with TMEM67 and CEP290 being the most frequently mutated genes [13]. Additionally, Itoh et al. identified a unique variant of CEP290 as the causative mutation in Arima syndrome [14]. Currently, there is no definitive cure for JSRD. Management focuses on symptomatic treatment, including ophthalmological, nephrological, respiratory, and plastic surgical interventions; behavioral and neurological support for developmental and autistic features, and comprehensive rehabilitation for psychomotor delays. Children with JSRD commonly exhibit intellectual disabilities and behavioural or emotional challenges [15]. However, rehabilitation approaches for JSRD remain underreported. In Japan, Mano et al. emphasized the importance of multidisciplinary rehabilitation, including physical therapy, occupational therapy, speech-language-hearing therapy, and orthotic interventions, to improve function and promote participation in daily activities [16]. As a culmination of efforts by the research group, the Clinical Practice Guideline 2018 for Joubert Syndrome and Related Disorders was published [11]. The guideline was developed by a multidisciplinary team, including pediatric neurologists and specialists in respiratory medicine, nephrology, plastic surgery, rehabilitation, and ophthalmology, reflecting the diverse clinical manifestations of the syndrome. Within Japan's designated intractable disease system, Arima syndrome was initially recognized and subsequently expanded to include JSRD in FY2018. The criteria for designated intractable diseases are as follows: (1) rarity (affecting < 0.1% of the population in Japan), (2) unknown etiology, (3) lack of effective treatment, (4) necessity of long-term treatment, and (5) existence of objective diagnostic criteria [17]. The Research Project on Treatment for Specified Diseases provides assistance for selected diseases among the designated intractable diseases that are entitled to the research grant program. The aspects of this project include research promotion, as well as financial assistance, to avoid catastrophic expenditure, as it covers the co-payment of eligible patients in return for providing data for research use [17].

In parallel with the research activities, preparations to establish a patient and family advocacy group for JSRD in Japan began in 2015, and the group was officially founded in 2016 [18]. Since then, patient and family meetings have been held approximately once a year, and the meeting was held in 2025 at our facility. Following the meeting, a questionnaire survey was conducted, and the families reported high satisfaction with the meeting [19]. At the same time, while organising the conference, it became clear that the unmet needs of patients and their families span a wide range of areas, including medical care, welfare, and education. While it has been reported that there are approximately 100 JSRD patients in Japan, as of the meeting date [11], 63 families were registered with the advocacy group. Although a survey conducted through the advocacy group may not be as comprehensive as a nationwide epidemiological survey, it could still produce similar results. This study aimed to assess the current situations, needs, and concerns about medical care, welfare, and education of patients and families with JSRD through patient and family groups in Japan.

## Materials and methods

The patient and family advocacy group meetings for JSRD in Japan were organized by the group and held at Shizuoka Children's Hospital, Shizuoka City, capital of Shizuoka Prefecture, Japan [19]. The meeting was conducted in a hybrid format, allowing both on-site and online participation. The meeting consisted of a medical lecture by a rehabilitation physician member of the JSRD clinical practice guideline development committee and an open forum for information exchange among patients and families. After the meeting, a survey was conducted to assess the patients' current situations and their families' concerns about medical care, welfare, and education. Study participants included all members of the JSRD advocacy group in Japan, including those who attended the advocacy group's annual meeting in person and online or were unable to attend. As of the meeting date, 63 families were registered with the advocacy group. This study was approved by the Ethics Committee of Shizuoka Children's Hospital (Approval number: R7-3).

The survey was administered online using Microsoft Forms (Microsoft® Corp., Redmond, WA). It was developed by the study author and distributed following the meeting. The questionnaires (Appendix) included the following categories: patient information (e.g., age, sex, and symptoms); method of meeting participation (on-site or online, or not attend); patient's social and educational context (e.g., school type); rehabilitation received; and topics the families would like more information about. The survey was shared with members of the advocacy group, and cooperation was requested through the group's communication channels. Responses were provided by family members. In cases where a family had more than one patient with JSRD, separate responses were submitted for each individual. Informed consent for the academic use of the data was obtained from all respondents via the online form. This survey was conducted at the same time as the previous survey, which focused on evaluating satisfaction with the patient and family group meeting [19]. Therefore, the study participants are the same as those in the previous report [19]. Information needs were measured using a 5-point Likert scale set as follows: 1 - Unrequired, 2 - Somewhat unrequired, 3 - Neither required nor unrequired, 4 - Somewhat required, and 5 - Required. A Likert scale is an ordinal scale; while the responses can be rated or ranked, the distance between responses is not measurable [20]. However, when analyzing responses on a Likert scale, parametric tests are considered to be sufficiently robust and to yield results that are nearly unbiased and within an acceptable range of the true value [20]. In this study, the analysis was conducted under the assumption that the Likert scale point was a parametric variable. The more the information is needed, the higher the average will be above 3; the less information needed, the lower the average will be below 3. Each Likert scale point of the topics the families would like more information about was analysed using a t-test under the hypothesis of a mean of 3. P-values < 0.05 were considered statistically significant. JMP® Student Edition 19.1.0 (SAS Institute Inc., Cary, NC) was used for all statistical analyses. P-values < 0.05 were considered statistically significant.

## Results

A total of 41 responses were collected, corresponding to individual patients. Of these, 9 (22%) patients participated on-site, 20 (49%) participated online, and 12 (29%) did not attend the meeting. Patients' ages ranged from 0 to 39 years (median age: nine years). Among the 41 patients, 19 (46%) were female, 21 (51%) were male, and the sex of one patient was unspecified due to lack of response. Reported complications included ocular symptoms (37 patients, 90%), renal symptoms (8 patients, 20%), hepatic symptoms (6 patients, 15%), respiratory symptoms (16 patients, 39%), finger symptoms (6 patients, 15%), and neurological symptoms (38 patients, 93%), particularly epilepsy in 5 (12%) patients, motor development delay in 38 (93%) patients, and cognitive and/or language development delay in 32 (78%) patients; multiple responses were allowed.

**Figure 1:**
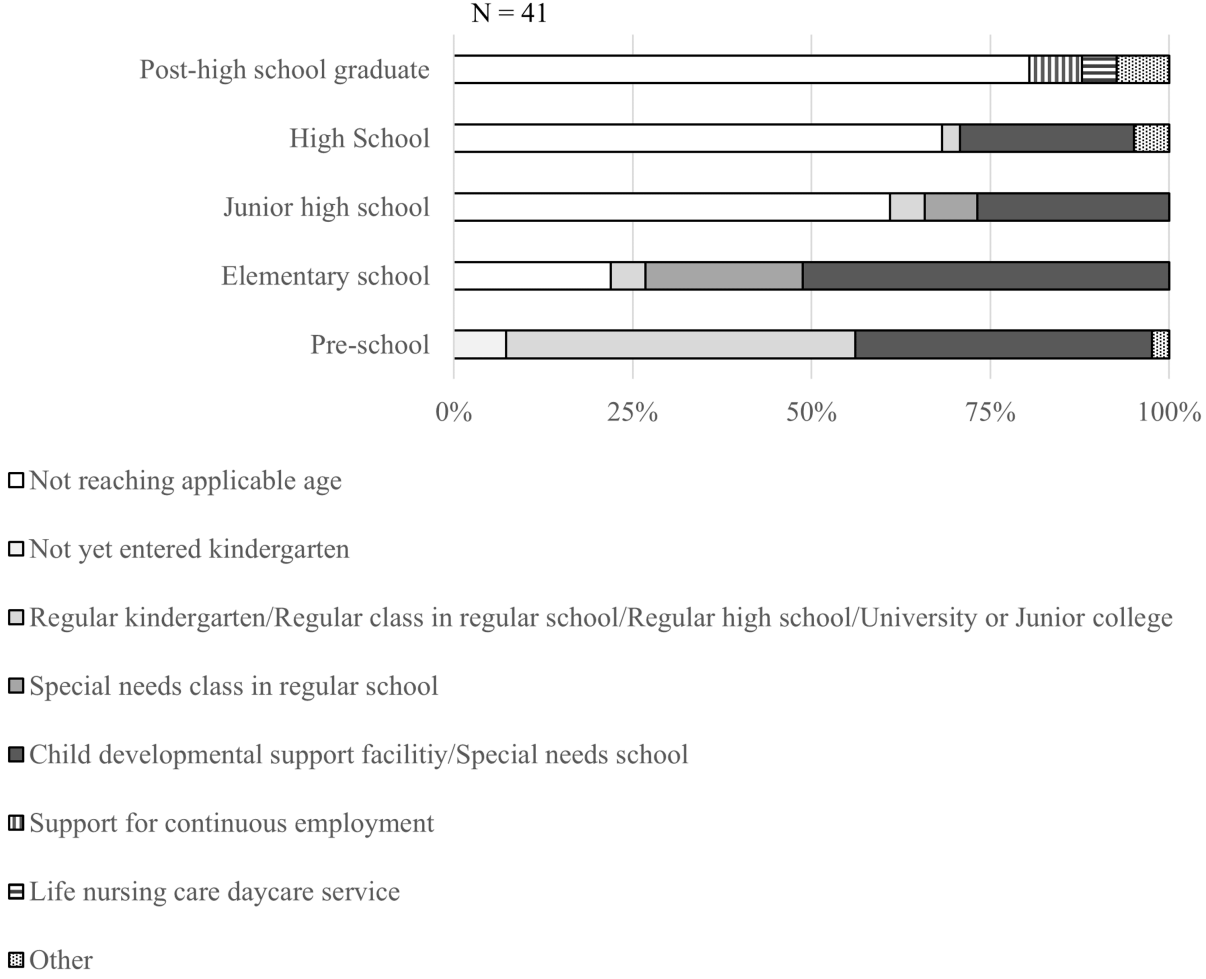
Patients' social situations Special educational support was provided to the majority of patients, in the form of special needs classes or school for those of school age.

Figure 1 summarizes the patient's current and previous social and educational placements (e.g., school type). In Japan, students with disabilities receive special instruction and support in various settings, including special needs schools (schools for children with comparatively severe disabilities), special needs classrooms (small classes for children with comparatively mild disabilities), and regular classrooms. Outside of school, welfare programs for those with special needs include child developmental support for primarily preschool-aged children, after-school day services during school years, and support for continuous employment after graduation. A progression-related trend was observed, with higher rates of special educational support reported as grade level increased. Many patients were enrolled in or had attended special needs schools or received individualized educational accommodations.

**Figure 2:**
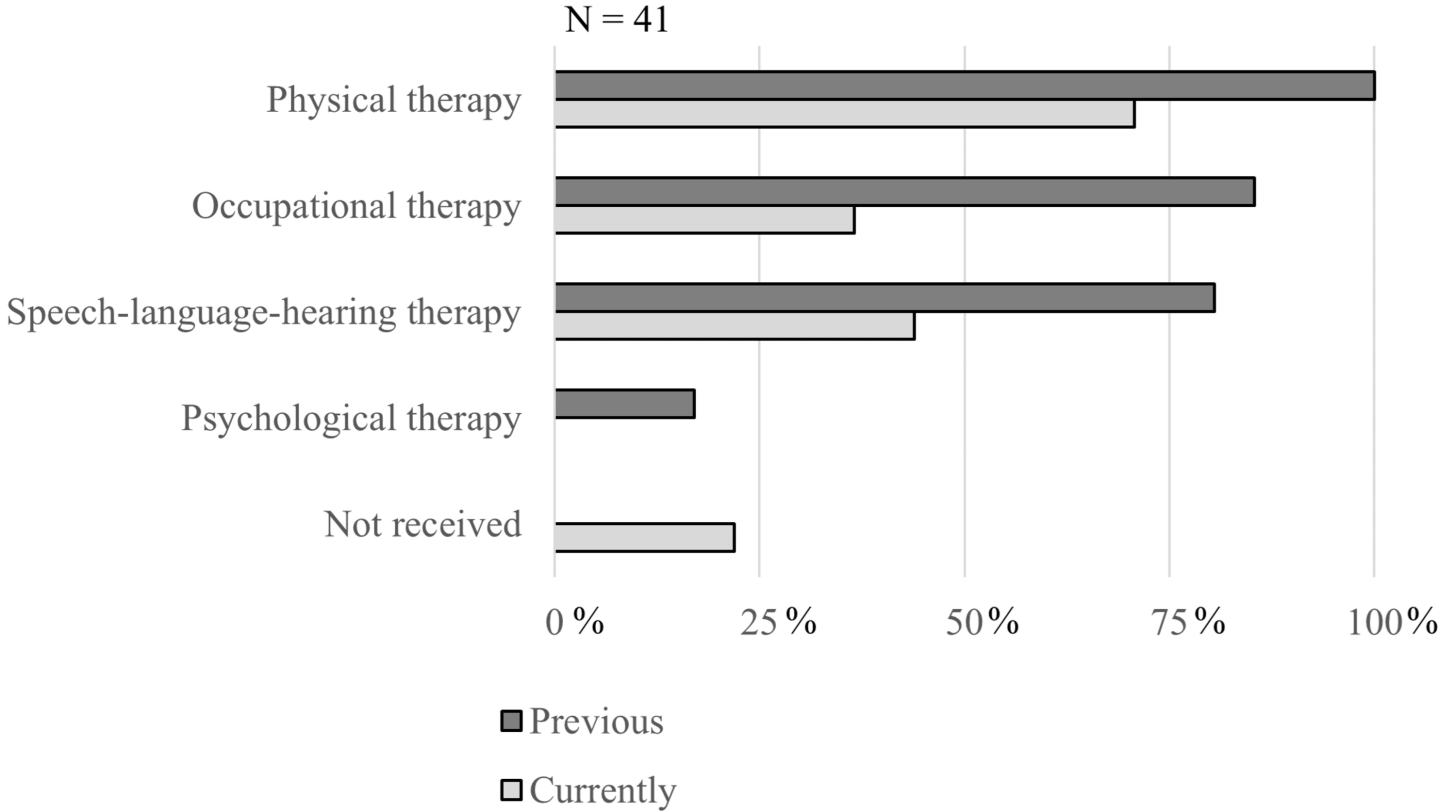
Current status of rehabilitation received All patients had received rehabilitation, and around three-quarters were currently undergoing it, while the remaining quarter were not.

Figure 2 shows the types and current status of rehabilitation received. All patients had received physical therapy, and many had also undergone occupational therapy and speech-language-hearing therapy. Among the 41 patients, 32 (78%) were actively receiving rehabilitation at the time of the survey, while the remaining nine (22%) were not.

**Figure 3:**
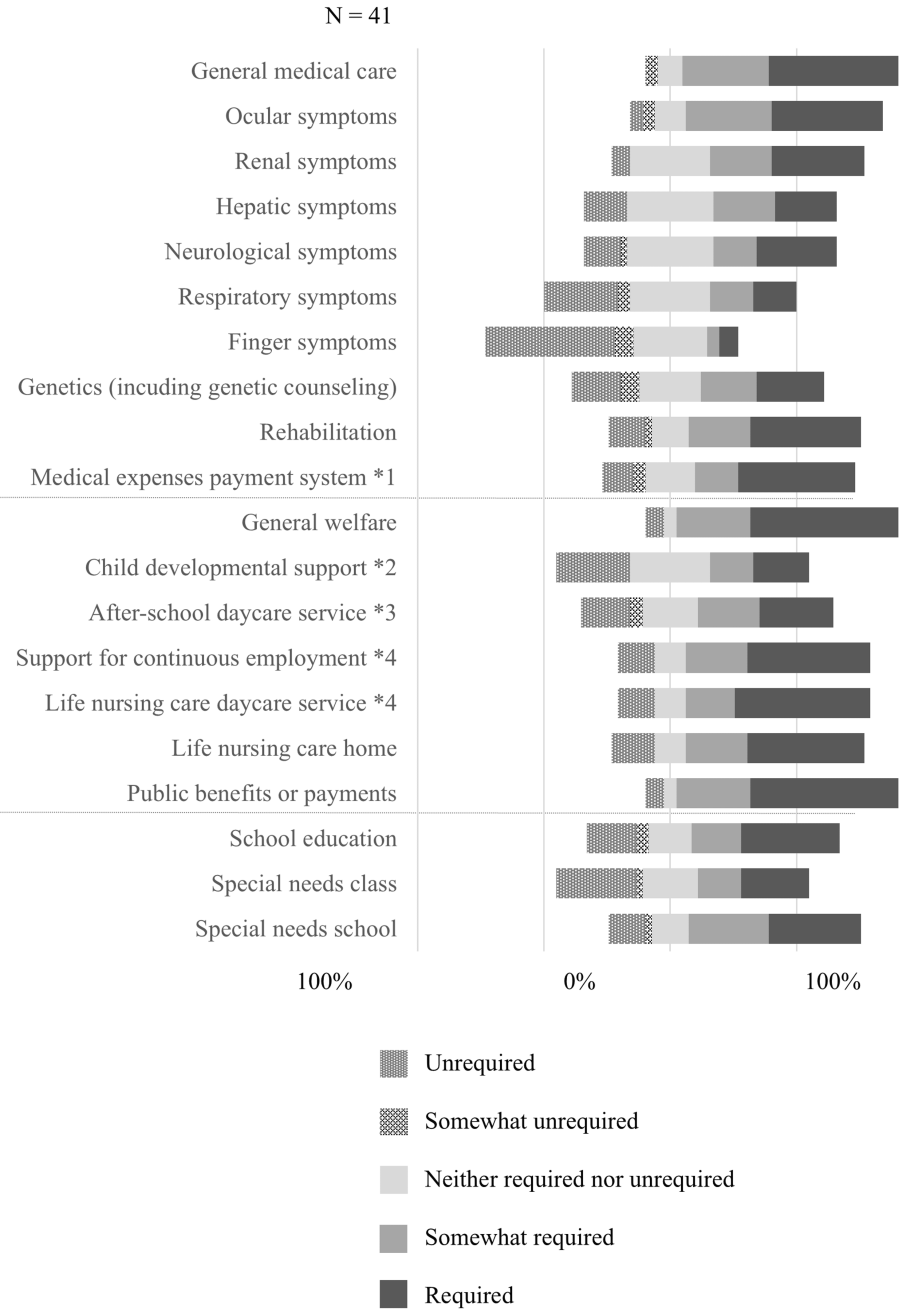
Information needs in the medical care, welfare, and education The need for information was high across numerous items in all domains, including health care, welfare, and education. *1: including the system for patients with designated intractable diseases; *2: support for pre-school children; *3: support for school-age children; *4: support for after the completion of school

Figure 3 highlights the types of information families wish to receive. Table 1 indicates the means of Likert scale points of items in the medical care, welfare, and education domains. Generally, the need for information was high across all categories. The majority of responses were "Required" or "Somewhat required" for most of the items. On the other hand, some responses - such as "Unrequired," "Somewhat unrequired," and "Neither required nor unrequired" - were also seen to some extent, depending on the items. Within the medical care domain, the means of Likert scales - that is, the demand for information - were significantly high in the items of general medical care, ocular and renal symptoms, rehabilitation, and the medical expenses payment system. In contrast, the value of finger symptoms was significantly low. Within the welfare domain, the means of Likert scales were significantly high in the items of general welfare, support for continuous employment, life nursing care, daycare service, life nursing care home, and public benefits or payments. Within the education domain, the means of Likert scales were significantly high in the items of school education and special needs school.

**Table 1:** Means of Likert scale points of items in the medical care, welfare, and education domains Likert scale scores were analyzed using a one-sample t-test under the null hypothesis that the mean was 3. P-values < 0.05 were considered statistically significant. N = 41.

Parameters	Mean of Likert scale points	t-value	p-value
General medical care	4.3	9.9	< 0.0001
Ocular symptoms	4.1	6.2	< 0.0001
Renal symptoms	3.8	4.6	< 0.0001
Hepatic symptoms	3.4	1.9	0.069
Neurological symptoms	3.5	2.3	0.027
Respiratory symptoms	2.9	-0.5	0.59
Finger symptoms	2.1	-4.4	< 0.0001
Genetics (incuding genetic counseling)	3.3	1.3	0.20
Rehabilitation	3.8	3.6	0.0008
Medical expenses payment system *1	3.8	3.7	0.0007
General welfare	4.3	7.6	< 0.0001
Child developmental support *2	3.0	0.1	0.92
After-school daycare service *3	3.4	1.7	0.096
Support for continuous employment *4	3.9	4.2	0.0001
Life nursing care daycare service *4	4.0	4.4	< 0.0001
Life nursing care home	3.8	3.6	0.0008
Public benefits or payments	4.3	7.6	< 0.0001
School education	3.5	2.2	0.031
Special needs class	3.0	0.2	0.85
Special needs school	3.7	3.4	0.0015

## Discussion

Families expressed a consistently high need for information across three main areas: medical care, welfare, and education. In the medical domain, the demand for information on ocular and neurological symptoms was especially high, reflecting the high prevalence of these complications among patients. In contrast, the need for information on finger abnormalities was relatively low, likely due to the lower frequency of these symptoms and the availability of well-established treatment protocols, such as surgical correction for polydactyly. While renal and hepatic symptoms were less commonly reported, the related need for information remained high. These symptoms may emerge or worsen over time and are often complex to manage, which may account for the elevated concern among families.

Similarly, the demand for information on rehabilitation was high. All patients had previously received rehabilitation therapy, with a significant proportion continuing therapy at the time of the survey. However, there is limited literature on rehabilitation and educational interventions specifically tailored for children with JSRD [21]. As part of the healthcare recommendations for JSRD, Bachmann-Gagescu reported that, to build on areas of strength and target areas of weakness, periodic developmental monitoring, along with standard physical, occupational, and speech-language-hearing therapies, and educational interventions, is required [22]. However, no specific types of therapy have yet been demonstrated to be more or less effective for this population. Peer support, including the exchange of information and updates on rehabilitation and education, would likely be beneficial for the families of patients. Patient group meetings of rare and intractable diseases can benefit families and provide healthcare professionals, including rehabilitation professions with valuable insights into the conditions and treatments associated with the disease.

In addition to the medical expenses payment system, within the welfare domain, there was a high demand for information related to post-graduation and public benefits. Similarly, information on special needs education services was highly sought after. Most patients had received educational support, including special educational needs provision. Given the nature of this rare and intractable disease, concern regarding medical expenses and financial support is to be expected. In Japan, JSRD is registered under both the Specific Chronic Pediatric Diseases Program and the Designated Intractable Disease Program, enabling patients to receive financial support for medical expenses. Patients and families may also be eligible for various welfare support systems, though eligibility often depends on the severity of the disability and the policies of the municipality in which they reside. The same applies to education services. In this perspective, peer support is considered valuable for learning about the experiences of other patients and families [23]. The need for information related to post-graduation was also high. This may reflect the increasing support requirements, including special needs education, as patients grow older, and the anticipated challenges they may face after completing school. Sharing the lived experiences of older patients through the advocacy group may be particularly helpful.

In Japan, the National Programme on Rare and Intractable Diseases began in 1972. The government, particularly the Ministry of Health, Labor, and Welfare, has promoted research and expanded support for individuals with rare and intractable diseases, including financial assistance. Government-funded research groups have investigated the prevalence, diagnosis, and management of these diseases, as well as patients' quality of life. These groups also serve as a network for disseminating diagnostic and treatment information throughout Japan [17,24]. Specific initiatives for JSRD in Japan have included epidemiological surveys, publication of clinical guidelines, registration as a designated intractable disease, and the establishment of patient and family associations. However, further efforts by experts in the fields of healthcare, research, and support systems are required to address the ongoing needs of patients with rare and intractable diseases and their families. The findings of this study could serve as a valuable evidence base for funding or policy advocacy recommendations, such as supporting continued or expanded funding for designated intractable disease programs.

As a limitation of this study, it should be noted that the study was conducted among the members of a patient and family advocacy group, not all individuals with JSRD in Japan. Therefore, there is selection bias in this study. The findings may not fully reflect the status of all patients. Further, this was a descriptive cross-sectional survey, and the questionnaire was not validated. Confounding factors, such as age and issues related to multiple comparisons, were also not evaluated in this study. Nationwide surveys on the prevalence and characteristics of rare diseases are often conducted in two stages in Japan [25,26]. In the first stage, the number of patients is surveyed among medical institutions; in the second stage, the characteristics of individual patients are surveyed based on the results of the first stage survey. A previous survey on JSRD in Japan was also conducted using the two-stage approach [11]. However, these research methods are time- and cost-consuming, which can be a significant challenge for many researchers. Through patient organizations, surveys on rare diseases can be conducted relatively easily to gather information on patients' situations and needs, but their validity must be carefully evaluated.

## Conclusions

This study involved a survey on the current status of patients with JSRD that was conducted through a patient and family advocacy group in Japan. Most patients with JSRD had received educational support, including special educational needs provision. Many patients undergo rehabilitation, with high demand and expectations for this treatment. The families reported significant demand for information across the domains of medical care, welfare, and education. Addressing the ongoing needs of patients and families with rare and intractable diseases in the areas of healthcare, research, and support systems remains a continuing challenge. Although a careful evaluation of validity is necessary, the survey conducted through patient and family advocacy groups for the rare diseases was relatively easy to implement and provided information about the patients' status and needs. The survey findings from the advocacy group could provide valuable evidence for funding or policy advocacy recommendations.

## Appendices

Questionnaire used in this study

The original Japanese questionnaire, which was administered online, relevant to this study, has been translated and reproduced by the author himself. Circles represent radio buttons (only one can be selected), and squares represent checkboxes (multiple responses are allowed).

1. How to participate in the patient and family advocacy group meeting this time

○ In-person participation

○ Online participation

○ Non-participation

2. Ages of the patient

(Enter a number)

3. Sex of the patient

○ Male

○ Female

○ Other

4. Patient's symptoms (multiple responses allowed)

□ Ocular symptoms

□ Renal symptoms

□ Hepatic symptoms

□ Neurological symptoms

□ Epilepsy

□ Motor development delay

□ Cognitive and/or language development delay

□ Respiratory symptoms

□ Finger symptoms

5. Patient's current and previous social and educational placements (select one main response)

5-1. Preschool

○ Not yet entered kindergarten

○ Regular kindergarten

○ Child developmental support facility

○ Other

5-2. Elementary school

○ Not reaching the applicable age

○ Regular class in a regular school

○ Special needs class in a regular school

○ Special needs school

○ Other

5-3. Junior high school

○ Not reaching the applicable age

○ Regular class in a regular school

○ Special needs class in a regular school

○ Special needs school

○ Other

5-4. High school

○ Not reaching the applicable age

○ Regular high school

○ Special needs school

○ Child developmental support facility

○ Other

5-5. Post-high school graduate

○ Not reaching the applicable age

○ University, junior college

○ Support for continuous employment

○ Life nursing care daycare service

○ Other

6. Status of rehabilitation (multiple responses allowed)

6-1. Rehabilitation services received to date

□ Not received

□ Physical therapy

□ Occupational therapy

□ Speech-language-hearing therapy

□ Psychological therapy

6-2. Rehabilitation services currently receiving

□ Not received

□ Physical therapy

□ Occupational therapy

□ Speech-language-hearing therapy

□ Psychological therapy

**Table 2:** Information required

Item	Required	Somewhat Required	Neither Required nor Unrequired	Somewhat Unrequired	Unrequired
General medical care	○	○	○	○	○
Ocular symptoms	○	○	○	○	○
Renal symptoms	○	○	○	○	○
Hepatic symptoms	○	○	○	○	○
Neurological symptoms	○	○	○	○	○
Finger symptoms	○	○	○	○	○
Genetics (including genetic counseling)	○	○	○	○	○
Rehabilitation	○	○	○	○	○
Medical expenses payment system	○	○	○	○	○
General welfare	○	○	○	○	○
Child developmental support	○	○	○	○	○
After-school daycare service	○	○	○	○	○
Support for continuous employment	○	○	○	○	○
Life nursing care daycare service	○	○	○	○	○
Life nursing care home	○	○	○	○	○
Public benefits or payments	○	○	○	○	○
School education	○	○	○	○	○
Special needs class	○	○	○	○	○
Special need school	○	○	○	○	○
